# Supplementary material for: Induction of cardiac fibulin-4 protects against pressure overload-induced cardiac hypertrophy and heart failure
Source: Commun Biol. 2025 Apr 24;8:661. doi: 10.1038/s42003-025-08087-8 (PMC12022050; doi:10.1038/s42003-025-08087-8)
Supplement: Supplementary file 2 — Supplementary Information [file 42003_2025_8087_MOESM2_ESM.pdf]

Table S1. Anatomical and functional data of fibulin-4$^{+/+}$ and fibulin-4$^{R/R}$ mice

	Fibulin-4$^{+/+}$	Fibulin-4$^{R/R}$
**Anatomical data**		
**Body weight (g)**	24.7 ± 0.8	23.8 ± 1.0
**Tibia length (cm)**	1.82 ± 0.01	1.80 ± 0.01
**LV weight (mg)**	89 ± 3	218 ± 16*
**RV weight (mg)**	22 ± 1	47 ± 4*
**Lung weight (mg)**	131 ± 2	162 ± 4*
**Heart weight / body weight (mg/g)**	4.5 ± 0.1	9.8 ± 0.7*
**Functional data**		
**Heart Rate (bpm)**	575 ± 5	555 ± 13
**MAP (mmHg)**	91 ± 2	69 ± 5*
**LV dp/dt$_{max}$ (mmHg·s^{-1})**	10770 ± 300	4650 ± 330*
**tau (ms)**	9.5 ± 0.8	13.4 ± 2.7
**Aorta parameters**		
**Aorta diameter (mm)**	1.44 ± 0.03	3.02 ± 0.14*
**Aorta distensibility (%)**	20.1 ± 1.5	12.2 ± 2.0*

Fibulin-4$^{+/+}$ (n=16), fibulin-4$^{R/R}$ (n=6), *P<0.05 vs fibulin-4$^{+/+}$, data is presented as mean ± SEM, a statistical t-test was performed. LV, left ventricle; RV, right ventricle; MAP, mean arterial pressure; LV dP/dt$_{max}$, maximum rate of rise of LV pressure; LVEDP, LV end diastolic pressure.

Table S2. Real-time PCR Primers used for mouse heart samples

Primer name	Sequence (5' --> 3')
**ANP FW**	TTCCTCGTCTTGGCCTTTTG
**ANP RV**	CCTCATCTTCTACCGGCATCTTC
**BNP FW**	GGGAGAACACGGCATCATTG
**BNP RV**	ACAGCACCTTCAGGAGATCCA
**αSKA FW**	TGACGTGTACATAGATTGACTCGTTT
**αSKA RV**	TGGCTGGCTTTAATGCTTCA
**SERCA2a FW**	GGTCAACGAGAGCACGGGGC
**SERCA2a RW**	GCCGGCAATTCGTTGGAGCC
**CTGF FW**	AGAACTGTGTACGGAGCGTG
**CTGF RV**	GTGCACCATCTTTGGCAGTG
**ELN FW**	GCCAAATACGGAGCCAGAGG
**ELN RV**	ACACCATAGCCAGGAAAGCC
**β-actin FW**	GGCACCACACYTTCTACAATG
**β-actin RV**	GGGGTGTTGAAGGTCTCAAAC

Table S3. Real-time PCR Primers used for human iPSC-derived cardiomyocytes

Primer name Taqman	assay number
**Fibulin-4**	Hs00973815_m1
**ANP**	Hs00383230_g1
**CTGF**	Hs00170014_m1
**PAI1**	Hs01126604_m1
**MYH6**	Hs01101425_m1
**MYH7**	Hs01110632_m1

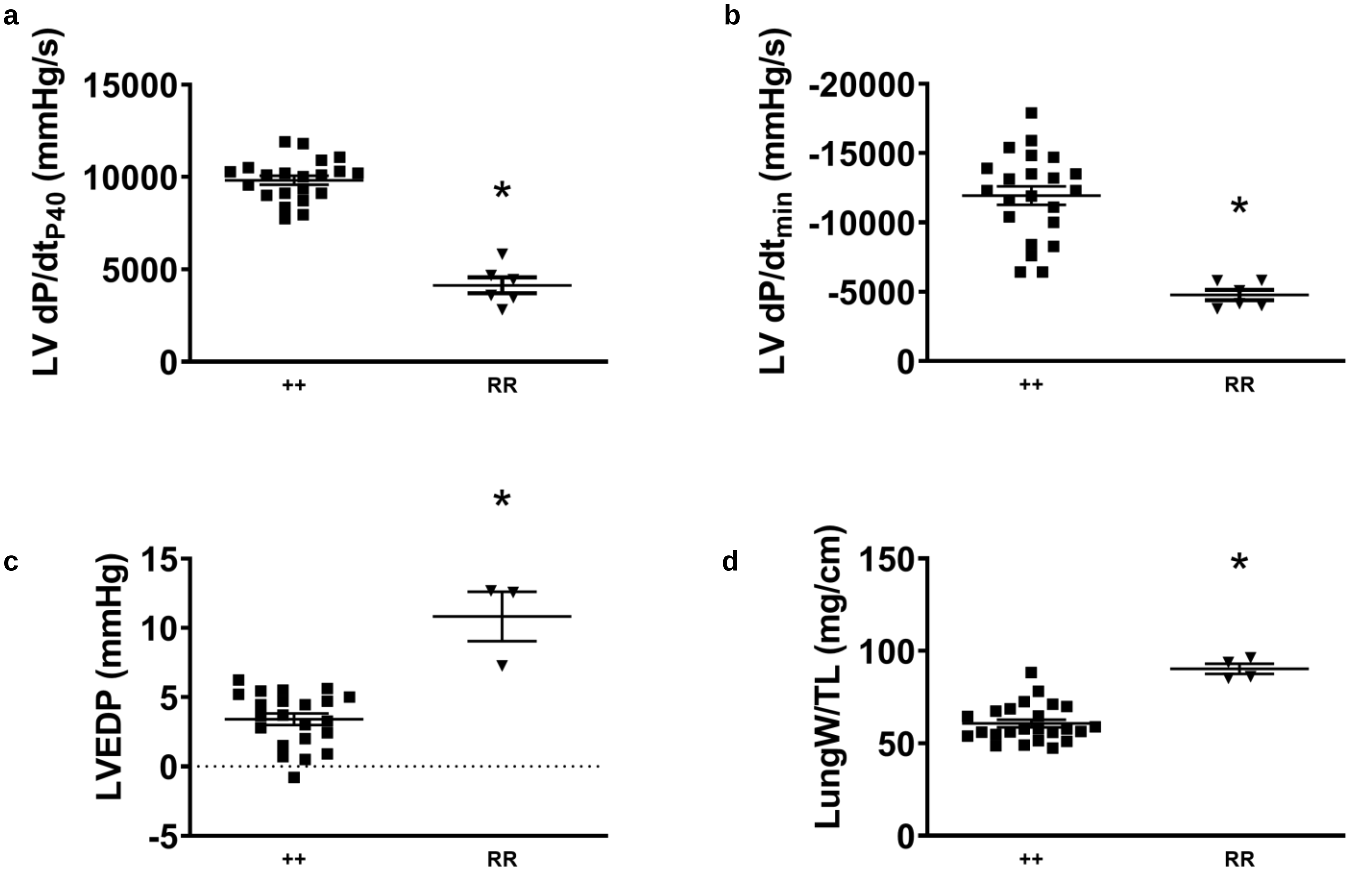

Fig. S1. Reduced fibulin-4 expression in fibulin-4$^{R/R}$ 18-week-old male mice, 4 weeks post-TAC, causes systolic and diastolic cardiac dysfunction as well as pulmonary congestion and aortic valve regurgitation.

LV dP/dt_{P40}, rate of rise in LV pressure at LV pressure of 40 mmHg; LV dP/dt_{min}, maximum rate of fall in LV pressure; LVEDP, left ventricular end diastolic pressure; LungW, lung weight; TL, tibia length. Fibulin-4$^{+/+}$ n=22, fibulin-4$^{R/R}$ n=6; *, $p<0.05$ vs fibulin-4$^{+/+}$. Lines represent mean ± SEM. One-way ANOVA was performed.

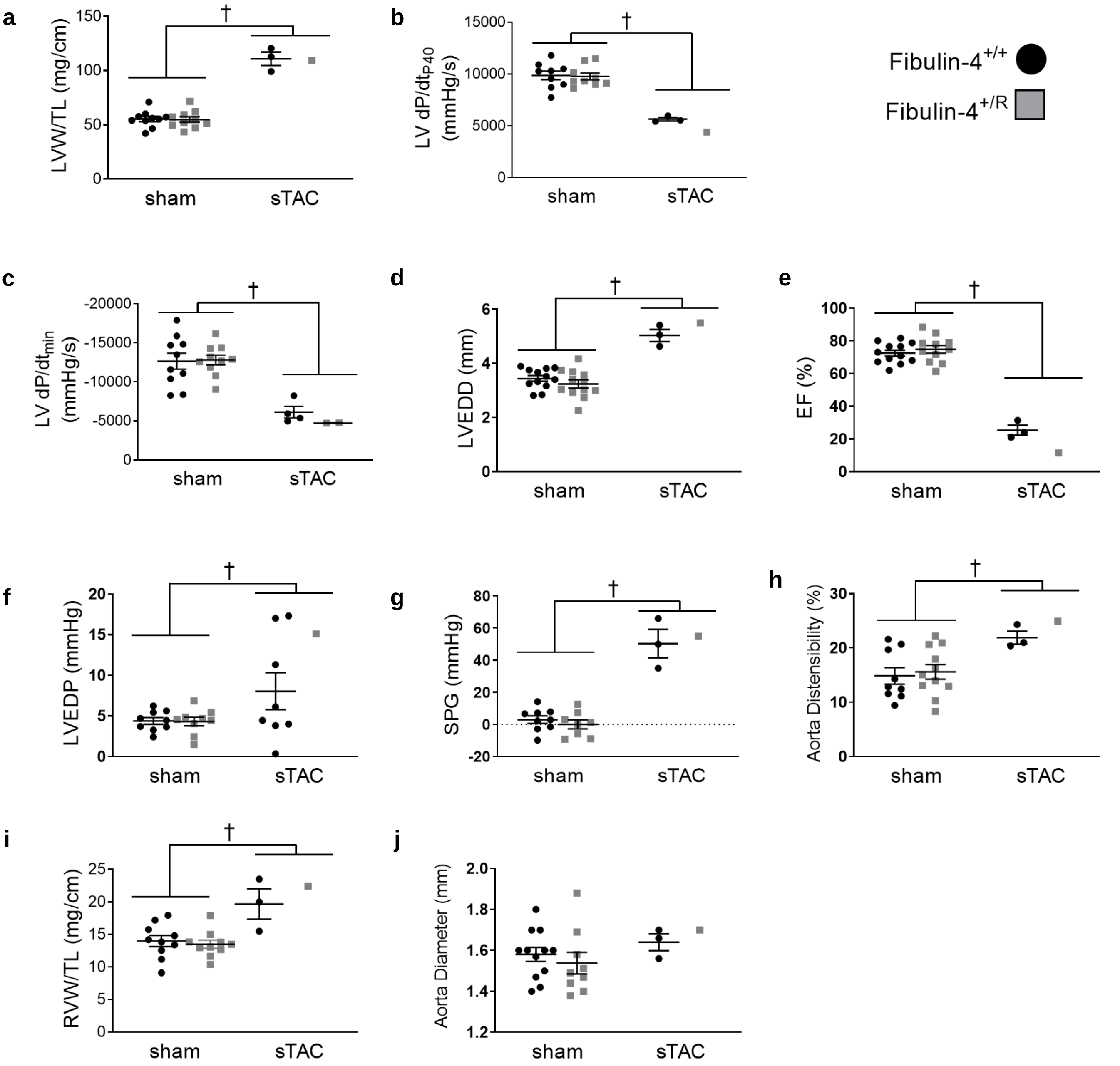

Fig. S2. Cardiac dimensions and function in sham operated and sTAC fibulin-4$^{+/+}$ and fibulin-4$^{+/R}$ 18-week-old male mice, 4 weeks post-TAC.

LVW, left ventricular weight; TL, tibia length; LV dP/dt$_{P40}$, rate of rise in LV pressure at LV pressure of 40 mmHg; LV dP/dt$_{min}$, maximum rate of fall in LV pressure; SPG, systolic pressure gradient; LVEDD, left ventricular end diastolic diameter; EF, ejection fraction; LVEDP, left ventricular end diastolic pressure; RVW, right ventricular weight. sham Fibulin-4$^{+/+}$ n=9, Fibulin-4$^{+/R}$ n=8; sTAC Fibulin-4$^{+/+}$ n=5, Fibulin-4$^{+/R}$ n=1 (all male). Lines represent mean ± SEM. One-way ANOVA was performed.

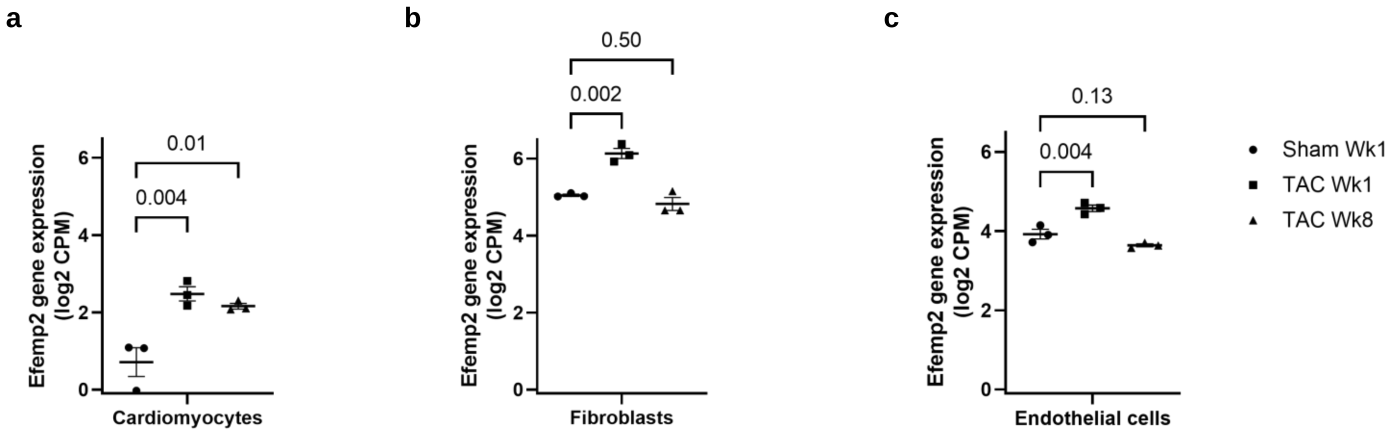

Fig. S3. Fibulin-4 gene expression increases and remains elevated in isolated cardiomyocytes after TAC.

RNA-sequencing data analyzed from the study of Froese et. al. [1]. 8-9 week old male C57Bl6N mice were subjected to TAC (26-gauge). Data represents *Efemp2* (fibulin-4) expression in log2 counts per million reads (CPM) in cardiomyocytes (a), fibroblasts (b) and endothelial cells (c) at 1 week (Wk) and 8 weeks of TAC. Lines represent mean ± SEM, n=3. One-way ANNOVA was used to determine significance.

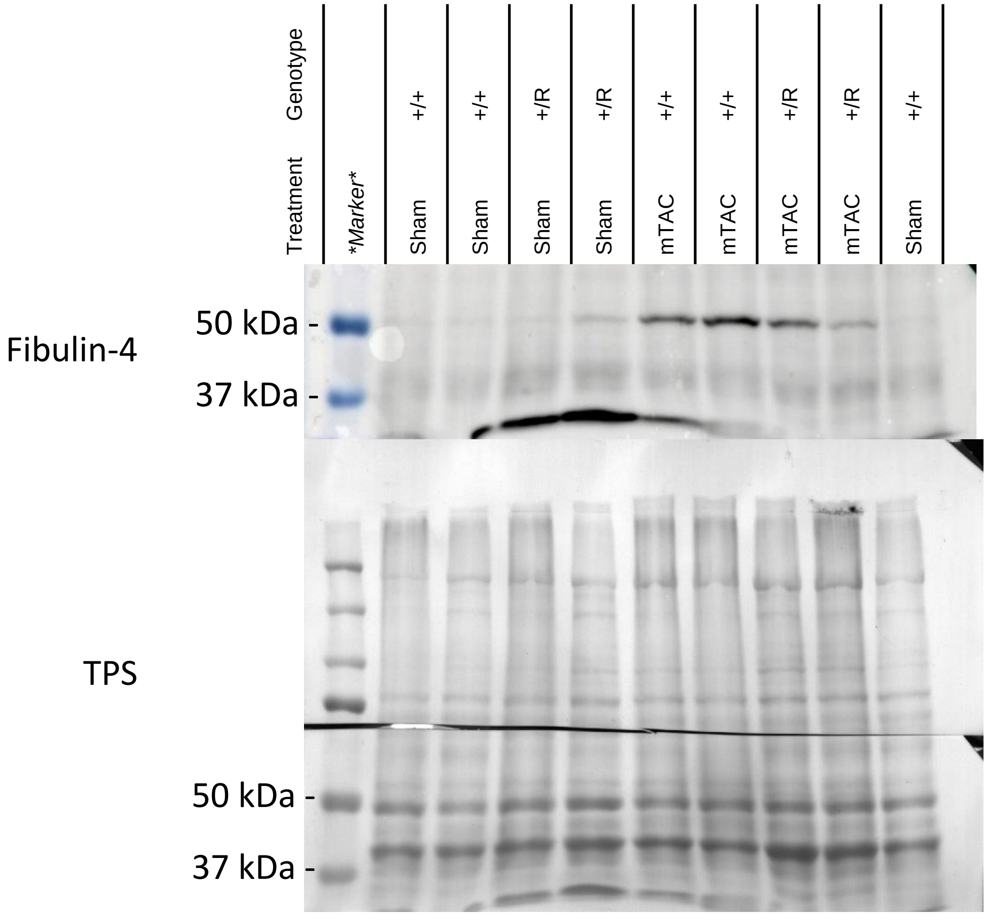

Fig. S4. Raw western blot images of fibulin-4 used in main Fig. 5.

TPS; total protein stain.

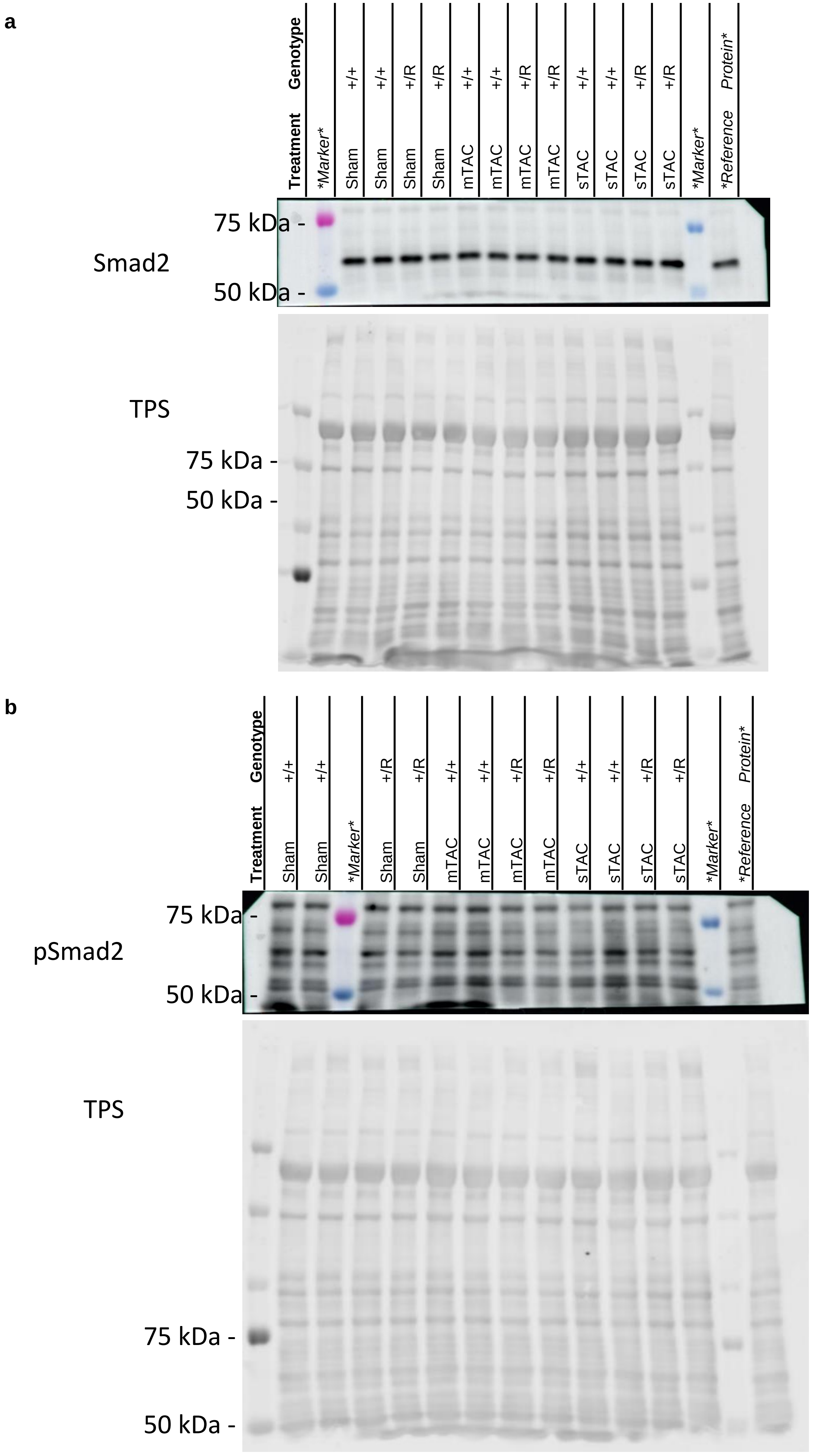

Fig. S5. Raw western blot images of Smad2 (a) and pSmad2 (b) used in main Fig. 5.

TPS; total protein stain.

a

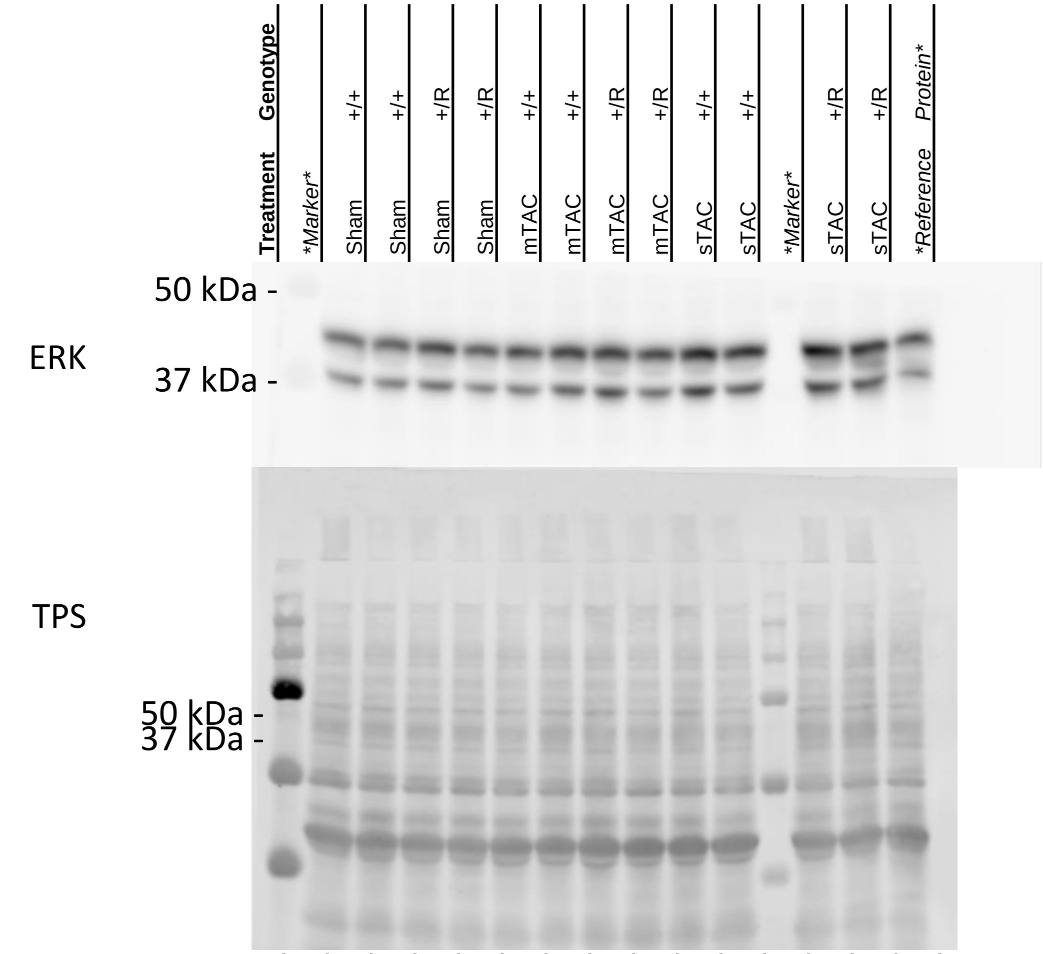

b

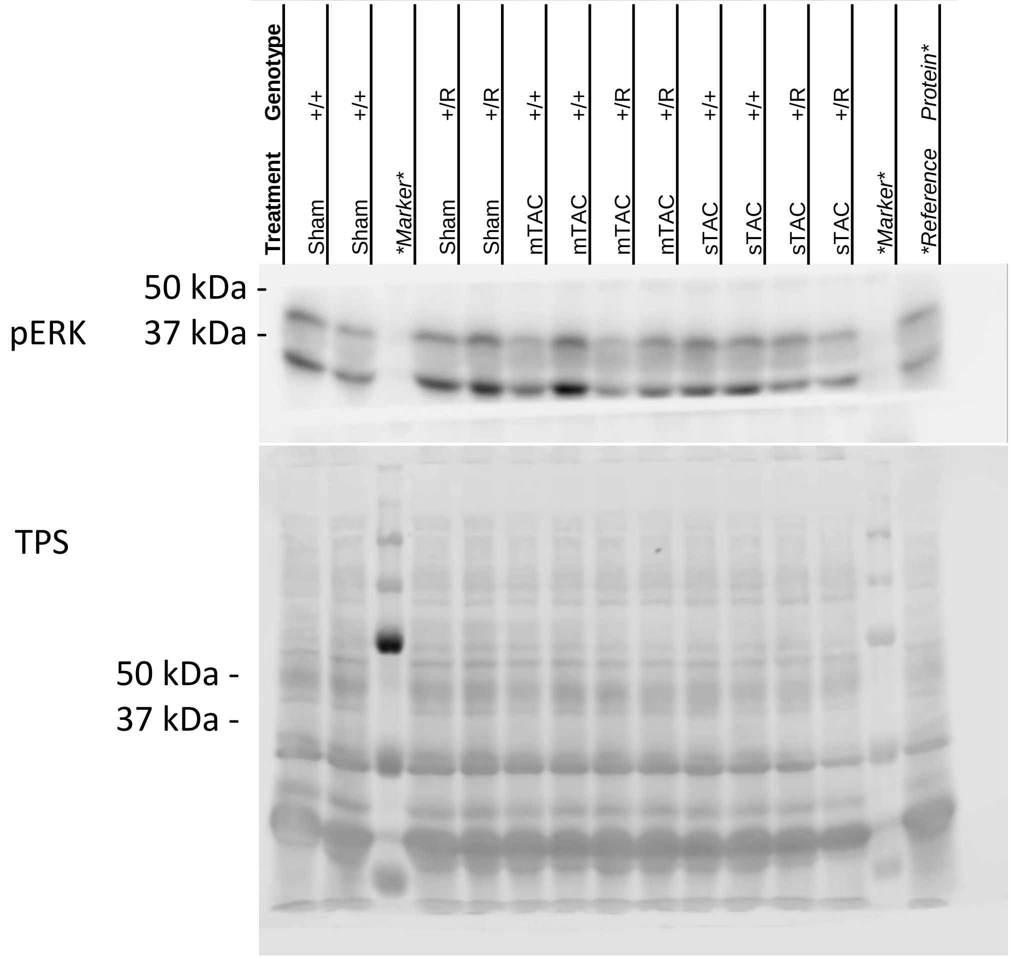

Fig. S6. Raw western blot images of ERK (a) and pERK (b) used in main Fig. 5.

TPS; total protein stain.

a

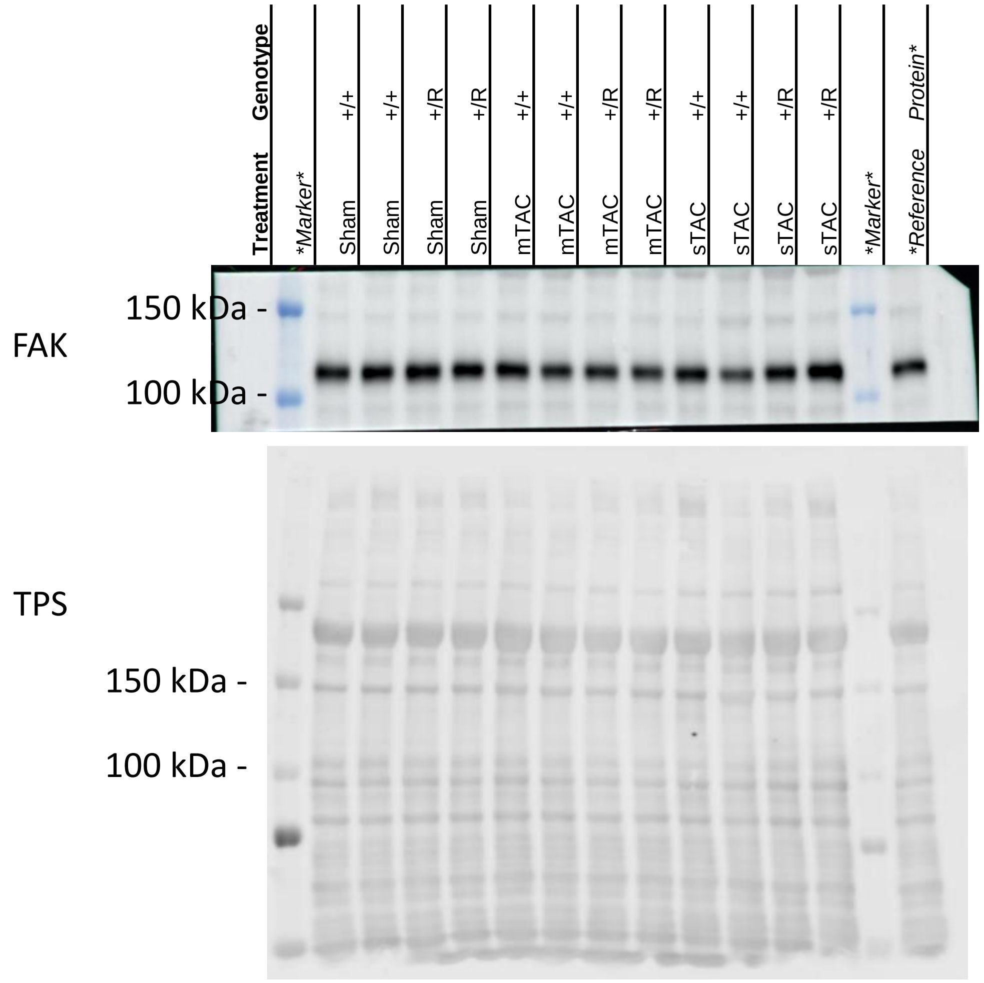

b

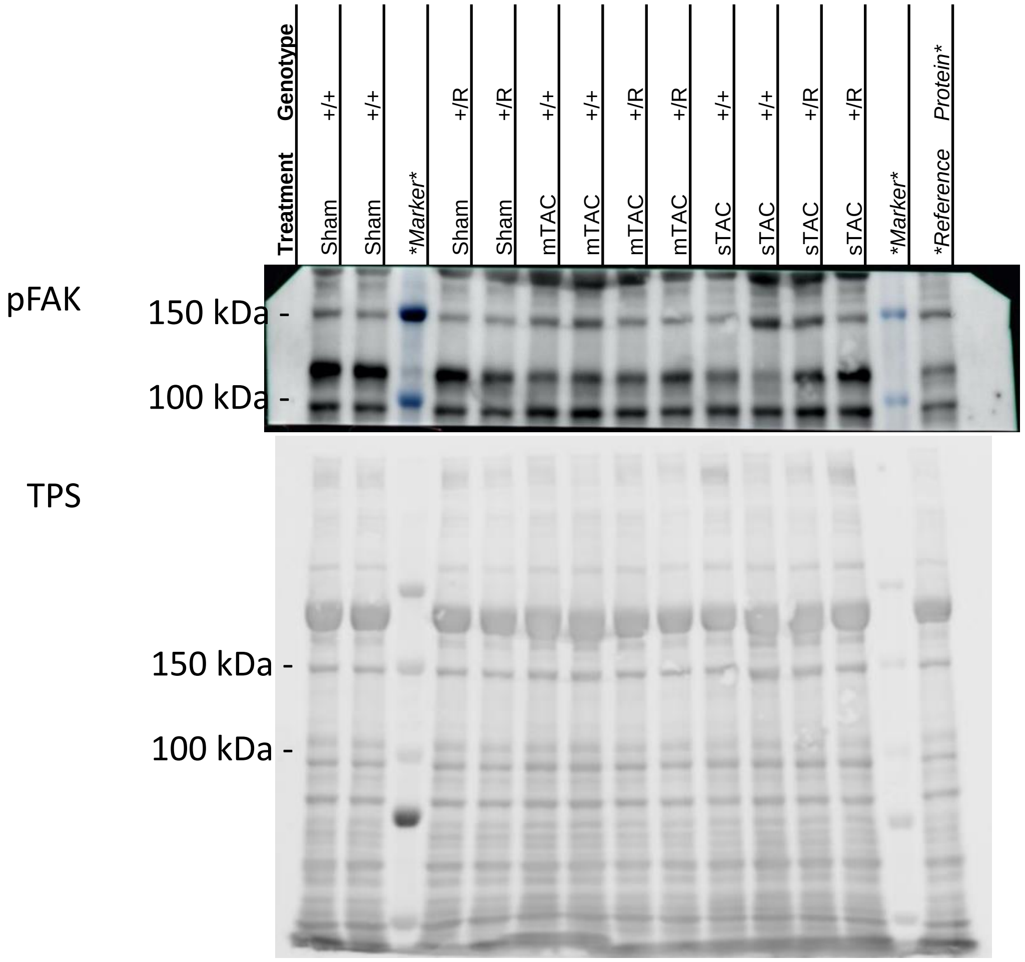

Fig. S7. Raw western blot images of FAK (a) and pFAK (b) used in main Fig. 5.

TPS; total protein stain.

Supplementary References

[1] Froese, N. *et al*. Analysis of myocardial cellular gene expression during pressure overload reveals matrix based functional intercellular communication. *iScience* **25**, 103965 (2022).
