# Supplementary material for: Induction of cardiac fibulin-4 protects against pressure overload-induced cardiac hypertrophy and heart failure
Source: Commun Biol. 2025 Apr 24;8:661. doi: 10.1038/s42003-025-08087-8 (PMC12022050; doi:10.1038/s42003-025-08087-8)
Supplement: Supplementary file 3 — Description of Additional Supplementary Files [file 42003_2025_8087_MOESM3_ESM.pdf]

Description of Additional Supplementary Files

File Name: Supplementary Data 1
Descriptions: Raw data of main Fig. 1-2.

File Name: Supplementary Data 2
Descriptions: Raw data of main Fig. 3-4.

File Name: Supplementary Data 3
Descriptions: Raw data of main Fig. 5-6.

File Name: Supplementary Data 4
Descriptions: Raw data of main Fig. 7.

File Name: Supplementary Data 5
Descriptions: Raw data of Supplemental Fig. S1-S2.
