# Supplementary material for: Induction of cardiac fibulin-4 protects against pressure overload-induced cardiac hypertrophy and heart failure
Source: Commun Biol. 2025 Apr 24;8:661. doi: 10.1038/s42003-025-08087-8 (PMC12022050; doi:10.1038/s42003-025-08087-8)
Supplement: Supplementary file 9 — Reporting Summary [file 42003_2025_8087_MOESM9_ESM.pdf]

nature portfolio

Corresponding author(s): J. Essers

Last updated by author(s): Mar 6, 2025

Reporting Summary

Nature Portfolio wishes to improve the reproducibility of the work that we publish. This form provides structure for consistency and transparency in reporting. For further information on Nature Portfolio policies, see our Editorial Policies and the Editorial Policy Checklist.

Statistics

For all statistical analyses, confirm that the following items are present in the figure legend, table legend, main text, or Methods section.

n/a	Confirmed	
☐	☒	The exact sample size (*n*) for each experimental group/condition, given as a discrete number and unit of measurement
☐	☒	A statement on whether measurements were taken from distinct samples or whether the same sample was measured repeatedly
☐	☒	The statistical test(s) used AND whether they are one- or two-sided<br>*Only common tests should be described solely by name; describe more complex techniques in the Methods section.*
☒	☐	A description of all covariates tested
☒	☐	A description of any assumptions or corrections, such as tests of normality and adjustment for multiple comparisons
☐	☒	A full description of the statistical parameters including central tendency (e.g. means) or other basic estimates (e.g. regression coefficient) AND variation (e.g. standard deviation) or associated estimates of uncertainty (e.g. confidence intervals)
☐	☒	For null hypothesis testing, the test statistic (e.g. *F*, *t*, *r*) with confidence intervals, effect sizes, degrees of freedom and *P* value noted<br>*Give P values as exact values whenever suitable.*
☒	☐	For Bayesian analysis, information on the choice of priors and Markov chain Monte Carlo settings
☒	☐	For hierarchical and complex designs, identification of the appropriate level for tests and full reporting of outcomes
☒	☐	Estimates of effect sizes (e.g. Cohen's *d*, Pearson's *r*), indicating how they were calculated

Our web collection on statistics for biologists contains articles on many of the points above.

Software and code

Policy information about availability of computer code

Data collection	no software was used
Data analysis	LV sections were stained with Picrosirius Red to assess collagen accumulation. Images were captured using commercial software (CLEMEX Vision and Visiopharm).

For manuscripts utilizing custom algorithms or software that are central to the research but not yet described in published literature, software must be made available to editors and reviewers. We strongly encourage code deposition in a community repository (e.g. GitHub). See the Nature Portfolio guidelines for submitting code & software for further information.

Data

Policy information about availability of data

All manuscripts must include a data availability statement. This statement should provide the following information, where applicable:

- Accession codes, unique identifiers, or web links for publicly available datasets
- A description of any restrictions on data availability
- For clinical datasets or third party data, please ensure that the statement adheres to our policy

access to primary datasets generated during the study are made available upon request

Research involving human participants, their data, or biological material

Policy information about studies with human participants or human data. See also policy information about sex, gender (identity/presentation), and sexual orientation and race, ethnicity and racism.

Reporting on sex and gender	n/a
Reporting on race, ethnicity, or other socially relevant groupings	n/a
Population characteristics	n/a
Recruitment	n/a
Ethics oversight	n/a

Note that full information on the approval of the study protocol must also be provided in the manuscript.

Field-specific reporting

Please select the one below that is the best fit for your research. If you are not sure, read the appropriate sections before making your selection.

☒ Life sciences ☐ Behavioural & social sciences ☐ Ecological, evolutionary & environmental sciences

For a reference copy of the document with all sections, see nature.com/documents/nr-reporting-summary-flat.pdf

Life sciences study design

All studies must disclose on these points even when the disclosure is negative.

Sample size	Sample size was determined based on an alpha of 0.05 and a power of 80% using a two-tailed test. A difference of 25% was expected between wild type and fibulin-4+/R littermates with a standard deviation of 18%. Based on this, we calculated the sample size to be N = 2*[(1.96+0.84)2 / (25/18)2] = 8.1 = 9 animals per group. We expected a 35% dropout due to heart failure as a result of the surgery, and a 5% dropout during surgery due to technical reasons. To keep 9 animals in the TAC groups, the size for these groups was set to 15 animals. In total, 354 animals were required (sham; 9 animals for 7 timepoints = 63 wild type and 63 fibulin-4+/R animals, TAC; 15 animals for 7 timepoints = 105 wild type and 105 fibulin-4+/R animals, no surgery; 9 animals for 2 timepoints = 18 fibulin-4R/R animals).
Data exclusions	no data exclusion
Replication	n/a
Randomization	allocation was done randomly
Blinding	Blinding was done where possible

Reporting for specific materials, systems and methods

We require information from authors about some types of materials, experimental systems and methods used in many studies. Here, indicate whether each material, system or method listed is relevant to your study. If you are not sure if a list item applies to your research, read the appropriate section before selecting a response.

Materials & experimental systems

n/a	Involved in the study	
☐	☒	Antibodies
☐	☒	Eukaryotic cell lines
☒	☐	Palaeontology and archaeology
☐	☒	Animals and other organisms
☒	☐	Clinical data
☒	☐	Dual use research of concern
☒	☐	Plants

Methods

n/a	Involved in the study	
☒	☐	ChIP-seq
☒	☐	Flow cytometry
☒	☐	MRI-based neuroimaging

Antibodies

Antibodies used	α-fibulin-4 (Rabbit polyclonal,Takako Eshashi, PMID: 17324935) , α-actinin (1:250, Sigma-Aldrich) antibodies, anti-Elastin (Abcam) secondary antibodies (Molecular Probes), α-myosin heavy chain (clone MF20, R&D Systems) fluorescently-labeled antibody, phospho-Smad2 (Ser465-467, Cell Signaling) or CTGF (GeneTex), vimentin (Cell Signaling), protein kinases 1 and 2 (pERK1/2) (Thr202/Tyr204) (Cell Signaling), ERK1/2 (Cell Signaling), phospho-focal adhesion kinase (pFAK) (Tyr397) (Invitrogen), FAK (Milipore) and SERCA2a (Abcam), α-myosin heavy chain (α-MHC) (clone MF20, R&D)
Validation	Validation according to manufacturer datasheet except for home-made fibulin-4 (personal gift Takako Eshashi, publication PMID: 17324935

Eukaryotic cell lines

Policy information about cell lines and Sex and Gender in Research

Cell line source(s)	iPSC-derived cardiomyocytes (iCell cardiomyocytes, CDI, Madison, WI, USA)
Authentication	by commercial supplier
Mycoplasma contamination	not tested for mycoplasm
Commonly misidentified lines (See ICLAC register)	n/a

Animals and other research organisms

Policy information about studies involving animals; ARRIVE guidelines recommended for reporting animal research, and Sex and Gender in Research

Laboratory animals	mice, C57Bl6
Wild animals	n/a
Reporting on sex	14-weeks old male fibulin-4+/+ and fibulin-4+/R mice were subjected to severe TAC (sTAC) (fibulin-4+/+ n=5, fibulin-4+/R n=5), or mild TAC (mTAC) (fibulin-4+/+ n=11, fibulin-4+/R n=17) using a 27G or 25G needle, respectively, or a sham operation (fibulin-4+/+ n=11, fibulin-4+/R n=10) as previously described
Field-collected samples	n/a
Ethics oversight	CCD/DEC consult , https://www.centralecommissiedierproeven.nl/

Note that full information on the approval of the study protocol must also be provided in the manuscript.

Plants

Seed stocks	n/a
Novel plant genotypes	n/a
Authentication	n/a
